# Assessing the influence of latency variability on EEG classifiers - a case study of face repetition priming

**DOI:** 10.1007/s11571-024-10181-2

**Published:** 2024-10-21

**Authors:** Yilin Li, Werner Sommer, Liang Tian, Changsong Zhou

**Affiliations:** 1 Department of Physics, Hong Kong Baptist University, Kowloon Tong, Hong Kong SAR, China; 2 Institute of Interdisciplinary Studies, Hong Kong Baptist University, Kowloon Tong, Hong Kong SAR, China; 3 Centre for Nonlinear Studies and Beijing-Hong Kong-Singapore Joint Centre for Nonlinear and Complex Systems (Hong Kong), Hong Kong Baptist University, Kowloon Tong, Hong Kong SAR, China; 4 Institute of Computational and Theoretical Studies, Hong Kong Baptist University, Kowloon Tong, Hong Kong SAR, China; 5 Life Science Imaging Centre, Hong Kong Baptist University, Kowloon Tong, Hong Kong SAR, China; 6 Department of Psychology, Humboldt-Universität zu Berlin, Berlin, Germany; 7 Faculty of Education, National University of Malaysia, Kuala Lumpur, Malaysia; 8 Institute of Systems Medicine and Health Sciences, Hong Kong Baptist University, Kowloon Tong, Hong Kong SAR, China

**Keywords:** ERP, Single trial, Trial-to-trial variability, Latency jitter, Latency shifts

## Abstract

Data-driven strategies have been widely used to distinguish experimental effects on single-trial EEG signals. However, how latency variability, such as within-condition jitter or latency shifts between conditions, affects the performance of EEG classifiers has not been well investigated. Without explicitly considering and disentangling such attributes of single trials, neural network-based classifiers have limitations in measuring their contributions. Inspired by domain knowledge of subcomponent latency and amplitude from traditional cognitive neuroscience, this study applies a stepwise latency correction method on single trials to control for their contributions to classifier behavior. As a case study demonstrating the value of this method, we measure repetition priming effects of faces, which induce large reaction time differences, latency shifts, and amplitude effects in averaged event-related potentials. The results show that within-condition jitter negatively impacts classifier performance, but between-condition latency shifts improve accuracy, whereas genuine amplitude differences have no significant influence. While demonstrated in the case of priming effects, this methodology can be generalized to experiments involving many kinds of time-varying signals to account for the contributions of latency variability to classifier performance.

**Supplementary Information:**

The online version contains supplementary material available at 10.1007/s11571-024-10181-2.

## Introduction

**Fig. 1:**
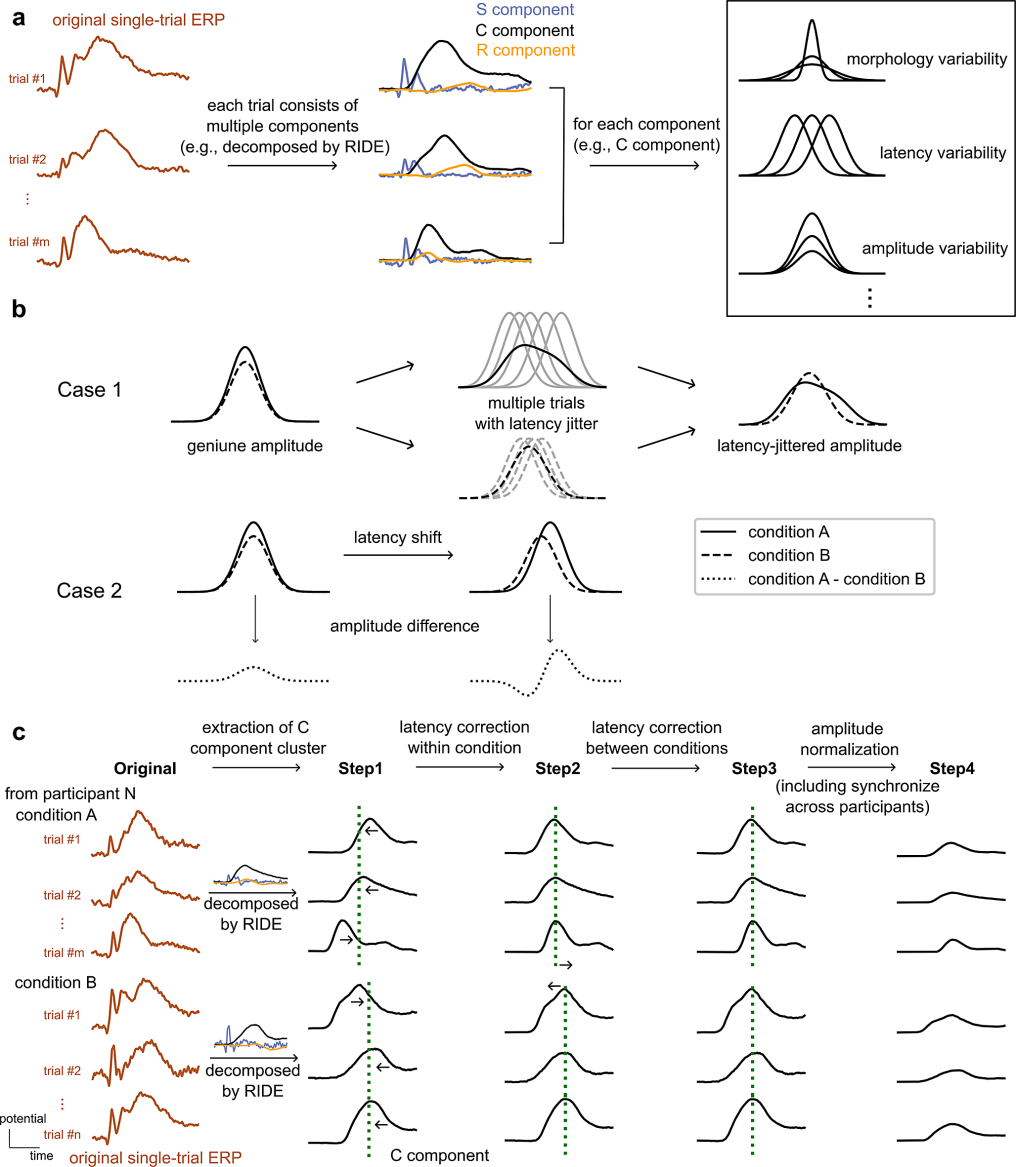
Illustration of the effects of trial-to-trial variability. **a** Single-trial event-related potentials (ERPs) commonly consist of multiple components, differing to some extent in morphology, latency, amplitude, and other parameters, including noise. **b** Latency variability can induce distortions in the averaged ERPs. Case 1: When multiple trials (gray curves, second panel) with latency jitter are averaged, the resulting amplitude is reduced and smeared out in time (third panel) compared to the true amplitude in each single trial (first panel). The degrees of distortion vary across different conditions. Case 2: Amplitude differences between conditions A (solid line) and B (dashed line) can arise from true amplitude differences (left panel), latency shifts, or both (right panel). **c** The pipeline of a stepwise latency correction method. Each curve representing potential over time corresponds to one single trial, illustrating the original ERP (Original) or C component (from Step1 to Step4). These trials exhibit distinct variability in latency, amplitude, and morphology. For illustration purposes, each subject exhibits three trials for each condition. In Step1, we utilize the residue iteration decomposition (RIDE) to decompose each original single-trial ERP into a stimulus-locked component S, central latency-variable component(s) C, and a response-locked component R. Then extract the C component cluster for further analysis. Step2 focuses on synchronizing the latency-variable C components within each condition for each participant to eliminate within-condition latency jitter. In Step3, we further synchronize trials across conditions based on latency lags between single trials and a new reference latency. Finally, in Step4, we synchronize trials across all participants and normalize amplitudes based on the global field power (GFP) per condition. The GFP is calculated as the standard deviation across all electrodes at each time point. The data at each step, as well as the original ERPs, are used as input for the DNN-based classifier to train different models. The purpose of these models is to predict the condition to which each single trial belongs (condition A or condition B). In this work, we considered primed and unprimed conditions in a face recognition task and employed EEGNet as the classifier

Event-related potentials (ERPs) are widely used for providing insights into the effects of different experimental manipulations or populations on neural activities. By comparing the averaged waveforms across different conditions or participant groups, researchers can identify their impact on neuro-cognitive processes and infer how the brain processes information, manifested as differences in amplitude, latency, or topography of specific ERP components (Luck 2014). These components occur within a short period around external events, mental processes, or behavioral responses. However, individual ERP components exhibit distinct physical characteristics, and the associated properties (e.g., amplitude, latency) may vary significantly from trial to trial, as depicted in Fig. 1a, which violates the assumption of homogeneity across trials when using the averaging technique (Stokes and Spaak 2016). The characteristics and variabilities of single trials may contain crucial information when interpreting neuro-cognitive processes (for a review, see Ouyang et al. 2017).

If not taken into account, trial-to-trial variability can lead to ambiguity of condition effects. In most cases, amplitude effects in averaged ERPs arise from a combination of genuine amplitude modulations and variabilities in latency. One source of latency variability is latency jitter between EEG signals within a given condition (Jung et al. 2001). As a consequence, the averaged ERPs may exhibit overlapping and blurred components, broadened shapes, reduced amplitudes, and other smeared properties, as demonstrated in Case 1 of Fig. 1b. Another type of latency variability is latency shifts between conditions or participant groups, as illustrated in Case 2 of Fig. 1b. Previous studies have consistently reported latency changes and reduced amplitudes in response to many cognitive tasks, or population differences as a function of maturation, aging, cognitive impairments, and neuro-cognitive disorders like Alzheimer’s disease (Rossion and Gauthier 2002; Pavarini et al. 2018; Paitel et al. 2021). Thus, multiple sources of latency variability can confound amplitude effects and preclude the condition effect’s interpretation from averaged ERPs.

In contrast to the canonical hypothesis-driven approach for measuring the attributes of components, such as the amplitude or latency of averaged ERPs at interested regions or intervals, recent data-driven strategies leverage deep neural networks (DNNs) as powerful models for analyzing features in EEG signals (for a review, see Bridwell et al. 2018). With only limited assumptions and constraints, DNNs are widely used as classifiers to discriminate between different conditions on a single-trial basis (Schirrmeister et al. 2017; Zhao et al. 2019; Roy et al. 2019; Xu et al. 2021; Gu et al. 2021; Yu et al. 2024). Due to the low signal-to-noise ratio of single trials and the data-driven strategy, DNNs do not explicitly identify or measure the amplitude and latency of signal voltages, and hence cannot distinguish between genuine amplitude differences and those induced by latency variations. Consequently, whether and how such basic properties as amplitude and latency of latency-varying signals contribute to classifier performance has not been well investigated.

This paper aims to incorporate domain knowledge of subcomponent latency and amplitude from conventional cognitive neuroscience into DNN exploration, guiding the identification of critical single-trial characteristics that influence classifier behavior. We proposed a novel stepwise latency correction approach for conditions and individuals, disentangling or controlling the effects of latency and amplitude variability on classifier performance. By systematically reducing different sources of variability, we effectively pinpointed genuine amplitude effects. Besides latency and amplitude, condition effects may also manifest in varying scalp distributions. Thus, the present study also examined the role of topographical distributions on classifier performance. To assess their effects, we analyzed changes in prediction accuracy, conducted temporal decoding analysis, and generated saliency maps. These metrics enabled us to quantitatively evaluate and visualize the influence of each factor on classifier behavior and associated cognitive processes.

We applied our analyses to the effects of repetition priming of learned faces as a case study. Humans are highly adept at recognizing faces, and this process becomes faster and more accurate when they are repeatedly presented with the same stimulus. Previous studies have identified at least two priming effects in ERPs to faces, namely the N250r and the N400, which are distinguishable in difference waves of the averaged ERPs between primed and unprimed conditions (Schweinberger et al. 1995; Schweinberger and Neumann 2016). However, another priming effect is also observed around 600 ms or later, which has rarely been taken into account in previous studies. This very late priming effect frequently exhibits a reversed polarity compared to the preceding N400 component. By utilizing our analytical pipeline, we obtained a deeper understanding of priming effects and how trial-to-trial variability can distort the associated condition effects and affect the classifier performance. Importantly, this approach can be applied to various scenarios beyond this specific case, providing a generalizable framework for understanding the interpretability of DNNs in cognitive neuroscience.

## Materials and methods

### EEG recording and preprocessing

In the original experiment (Nowparast Rostami et al. 2017), 211 healthy adults between the ages of 18 and 40 were tested. EEG signals were recorded from 42 Ag/AgCl electrodes, with the left mastoid electrode (A1) serving as the initial reference and the ‘AFz’ electrode as the ground. The EEGLAB toolbox was used for preprocessing. Blinks and eye movements were eliminated in the first step using independent component analysis (function: runica(); algorithm: Infomax (Gradient)). We used SASICA (an EEGLAB plugin) as a guideline for selecting and excluding artifact components. After filtering with a low-pass Hamming windowed sinc FIR filter with cutoffs at 0.1 and 40 Hz, the data were recalculated to an average reference. Trials with missing or erroneous responses, RTs shorter than 200 ms, or those identified as outliers by Tukey’s outlier filter were also eliminated, leaving 194 participants as the final sample ($\:{M}_{age}=27.8$ years with $\:{SD}_{age}=5.3$; 95 females). These trials were then downsampled to 250 Hz.

## Experimental paradigm

**Fig. 2:**
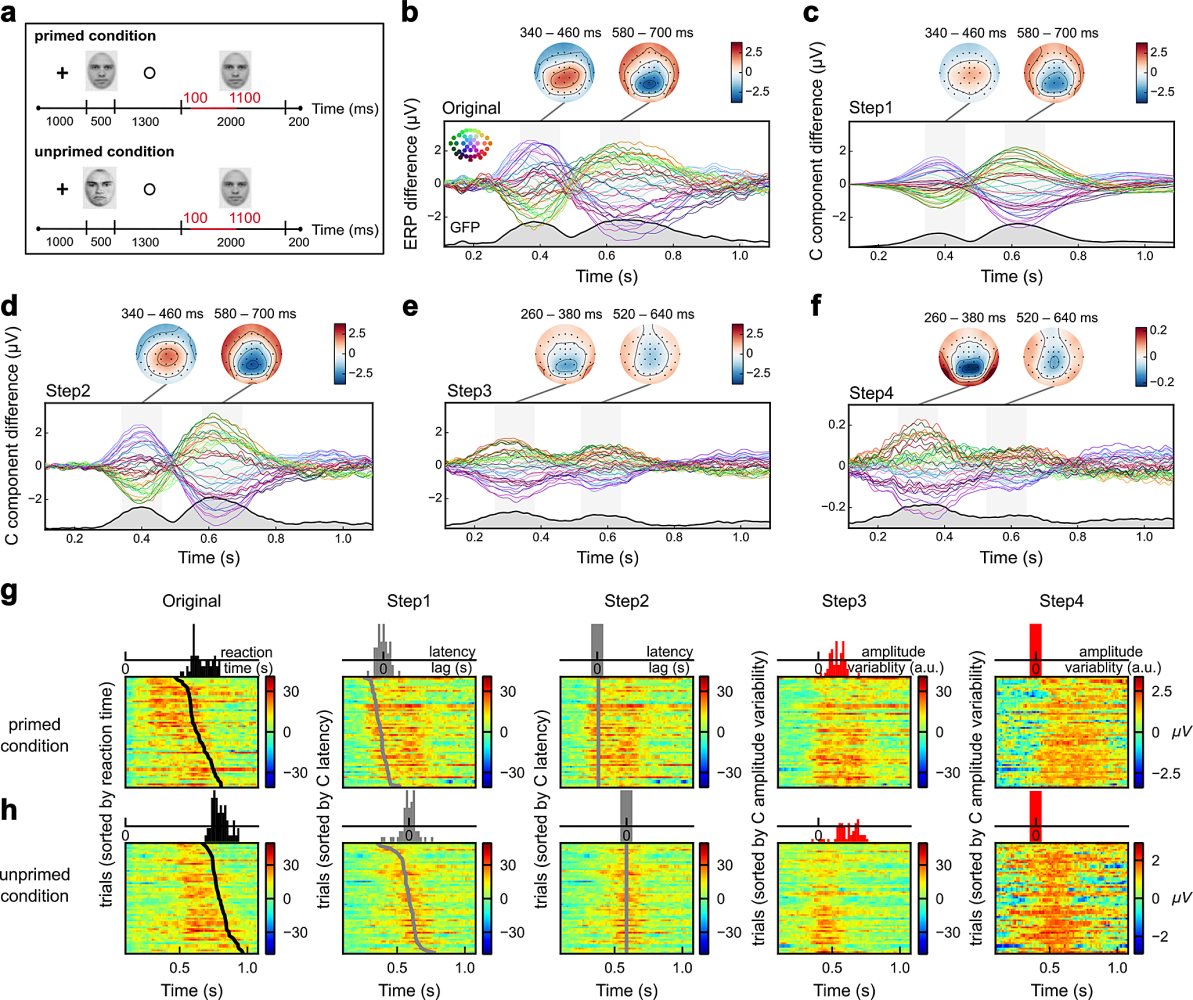
The latency variability confounds condition effects. **a** Trial scheme of the priming task with two conditions. In the primed condition, the two subsequent stimuli are identical faces, whereas in the unprimed condition, the prime stimulus differs from the target stimulus. Each trial consists of a 1000-ms fixation cross, a 500-ms prime stimulus, a 1300-ms fixation circle, and a 2000-ms target stimulus, followed by a 200-ms inter-trial interval. We selected the period from 100 ms to 1100 ms after the target stimulus onset for further analyses, represented by the red lines. **b-f** The original priming effects and after different steps of latency correction are shown by averaged difference waveforms between primed minus unprimed conditions. Each curve represents a different channel (inset). The bottom curve in each box represents the global field power (GFP), that is, the standard deviation across all electrodes for each time point. The shaded areas and corresponding topomaps illustrate the priming effects during two intervals (340–460 ms and 580–700 ms) from Original to Step2. Notably, these two effects exhibit reversed polarities. After synchronizing latency shifts between conditions, the periods of 260–380 ms and 520–640 ms are used to highlight different priming effects (Step3 and Step4). The reversed polarities disappear and latency-corrected amplitude differences are revealed. On the magnitude scale of normalization, the temporal region still shows positivity while the parietal area displays negativity. When further comparing the topomaps between these two intervals, we observed that the priming effect between 260 and 380 ms exhibits a larger parietal amplitude difference compared to the following priming effect (520 to 640 ms). The difference topomaps of GFP-scaled waveforms suggest that the activation level fluctuates based on this heterogeneity which indicates that primed and unprimed conditions indeed have different topographies over time. If they were the same, scaling would eliminate any difference. **g** The decomposed and synchronized results are visualized (for the ‘Pz’ electrode) by sorting the trials of one participant from the primed condition according to reaction times, latency lags, and amplitude variabilities. The axis scale from Original to Step3 is identical, while it was adjusted for Step4. **h** The results of sorting trials of the same participant from the unprimed condition

The original study employed a repetition priming paradigm to explore the effects of familiarity (familiar vs. unfamiliar) and difficulty (easy vs. difficult) on face and object (house) stimuli. In our data analysis, we only considered the tasks with familiar faces as targets in the easy conditions. The procedure and related data treatment were described in detail by Nowparast Rostami et al. (2017). Briefly, the experiment consisted of learning phases and recognition phases. During the learning phase for faces in the easy conditions, participants learned 12 initially unfamiliar faces. In the recognition phase, as illustrated in Fig. 2a, EEG signals were recorded using a prime-target paradigm. A prime stimulus was presented, which could either be identical to (primed condition) or different from (unprimed condition) the subsequent target stimulus; in the unprimed condition, the prime stimulus was an unfamiliar face, while in the primed condition, it was the same face as the target that had been learned earlier. Participants were required to indicate, by pressing a button as accurately and quickly as possible, whether the target stimulus was one of the learned faces. Each trial consisted of the presentation of a 1000-ms fixation cross, a 500-ms prime stimulus, a 1300-ms fixation circle, and a 2000-ms target stimulus, followed by a 200-ms inter-trial interval. Each condition – primed familiar or unprimed familiar faces – was realized in 72 trials. We chose epochs ranging from 100 ms before to 1500 ms after the target stimulus onset for decomposition. However, to avoid any potential influence on classification caused by zero padding from the synchronization process, we extracted epochs that ranged from 100 ms to 1100 ms post-target onset for subsequent analysis (as indicated by the red lines in Fig. 2a).

## EEGNet assessment of condition effects

In this paper, we used EEGNet as the classifier, which is a CNN architecture specifically designed for analyzing EEG single trials (Lawhern et al. 2018). It is capable of effectively learning from limited datasets, typically consisting of a few hundred trials per individual. The inputs to the model are single trials presented as 2D matrices, representing the voltages recorded at different channels over a long or short time. The model comprises several blocks.

1. The first block in the model is specifically designed for temporal convolution. Each temporal kernel convolves across the entire time series and all electrodes within a single trial. In this block, different kernels ($\:{K}_{1}$) can bandpass-filter the EEG signals into different frequency bands (outputs called feature maps), while the size of each kernel $\:(1,{\:L}_{1})$ can vary based on the input length, which is based on the whole epoch or sliding window for temporal decoding.
2. The second block is designed for spatial convolution. It summarizes information across all channels by applying a spatial convolution to each time point and all channels collectively. The size is $\:(C,\:\:1)$ where $\:C$represents the number of electrodes. Within each frequency band, there are $\:{K}_{2}$ spatial kernels. Consequently, the block utilizes a total of $\:{K}_{1}*{K}_{2}$ spatial filters, aiming to extract spatial information across different frequency ranges. To extract broader global information, we apply the average pooling layer following the exponential linear unit (ELU) activation function.
3. The third block is designed to decouple and integrate information across different frequency bands. Following the previous block, we obtain a total of $\:{K}_{1}*{K}_{2}$ feature maps. To explicitly separate the relationships between these feature maps, we learn a kernel of size $\:(1,{\:L}_{2})$ for each feature map. The length of $\:{L}_{2}$ is half of $\:{L}_{1}$. Subsequently, all the feature maps are merged using kernels of size $\:({K}_{1}*{K}_{2},\:\:1,\:\:1)$. This kind of pointwise convolution allows for optimal coupling of the previously obtained features, facilitating the integration of information across different frequency bands. Following this block, the features are flattened and the model outputs the predicted probability of belonging to each condition for the input signal.

During the training process, the weights of 8 kernels were optimized automatically through backpropagation, consisting of $\:{K}_{1}=4$ temporal kernels and $\:{K}_{2}=2$ spatial kernels for each temporal kernel. The length of the input varied depending on the specific analysis method employed. For general classification and further decoupling, we used the whole 1-second epoch, while for temporal decoding analysis, we used a shorter interval of 0.12 s. Regarding temporal decoding, it is important to consider that some ERP components are only of short duration. In such cases, employing long sliding windows could result in blurring, making shorter windows more suitable. Conversely, for phenomena with longer durations, both long and short sliding windows may be similarly effective in capturing the necessary temporal information. Additionally, all $\:C=41$ electrodes were included. The predicted probability was passed through a sigmoid function to calculate the categorical cross-entropy loss between the output and the target. We employed the Adam optimizer to train the model, aiming to minimize this loss function, with the learning rate set at 0.05. To accelerate model convergence, we applied batch normalization after the convolution operation in each block. Additionally, to prevent overfitting during training, we used dropout and weight decay, with a dropout rate of 0.2 and a weight decay of 0.001. The 10-fold cross-validation was employed to avoid overfitting and assure generalization abilities. The training and testing datasets were composed of 90% and 10% of the total data, respectively, across all participants and conditions. We evaluated the classifier performance by measuring the prediction accuracy on the testing dataset. A higher prediction accuracy indicates larger explicit or implicit differences between conditions.

## A stepwise latency correction model

As explained above, there are several types of trial-to-trial variability potentially blurring condition effects. To tease apart the potential influences of various factors on classifiers, we have devised a four-step pipeline (as illustrated in Fig. 1c) to effectively decompose and disentangle the related effects. We first decomposed the original single-trial ERPs (*Original*) into distinct component clusters (*Step1*), then synchronized the latencies of cognition-related component clusters to address within-condition jitter (*Step2*) and latency shifts between conditions (*Step3*). In this way, we can ultimately focus on the genuine effects on amplitude and spatial distributions (*Step4*). Next, we provide a comprehensive and detailed description of the latency correction model.

*Step1—extraction of C component cluster*. Considering that single trials consist of multiple components with varying latencies, a suitable method for latency correction should address both the decomposition of components and the estimation of their latencies simultaneously. To tackle this, we employed the residue iteration decomposition (RIDE) method (Ouyang et al. 2011, 2015a, b). RIDE can decompose the original single-trial ERPs into several component clusters across all channels. Typically, one can extract (a) a stimulus-synchronized component cluster (S component), reflecting sensory and perceptual processes; (b) a response-synchronized component cluster (R component), encompassing motor-related processes; and (c) an intermediate set of latency-variable components (C component), associated with central cognitive processes like stimulus classification or response selection. While the latencies of S and R components are determined and locked by stimulus onset and reaction time, respectively, the latency of C component is unknown. RIDE employs an iterative approach to generate and update spatiotemporal templates for each component cluster, which are the averaged latency-locked single trials. Since RIDE is applied on a per-subject, per-condition basis, the resulting template is unique to each subject, reflecting its own morphology, as well as spatial and temporal dynamics. The relative latency of C component in each single trial is determined by finding the maximum value of the time-lagged correlation coefficients between the trial and its template. After applying RIDE, we obtained all S, C, and R components for each trial, along with their corresponding relative latencies. In the current paradigm investigating high-level cognitive processes (face recognition), we prioritized the cognition-related components present in single trials by retaining the C components for further analysis while excluding the S and R components.

*Step2—latency correction within condition*. After decomposing single trials into different component clusters, we retained the cognition-related C components for further analysis. One source of variability is latency jitter, which can obscure components by introducing artificial changes in amplitude. Through the implementation of RIDE, we obtained the latency lags between each trial and its corresponding template. For instance, if there is a 50 ms latency lag, it signifies that the overall pattern of this trial occurs 50 ms later than the template’s pattern. To correct for jitter, we synchronized single trials by shifting them in the opposite direction, such as shifting the trial forward by 50 ms. This step effectively aligns the latency jitter within each condition while preserving the overall latency differences between conditions and participants.

*Step3—latency correction between conditions*. Latency shifts can introduce ambiguity in determining whether a specific process induces only amplitude differences, spurious amplitude differences induced by latency shifts, or a combination of both. Given that RIDE is applied on a per-subject and per-condition basis, and jitter correction is based on relative latency lags, comparisons between different conditions require the actual latency relative to the stimulus. To achieve this, we selected the peak latency of a specific electrode in the C component template as a reference for each condition and participant. Based on the median peak latency from templates of two conditions, we then synchronized the peak latencies of all trials per participant. In this way, the impact of between-condition latency shifts is effectively eliminated or at least strongly reduced.

*Step4—amplitude normalization*. After Step3, while latency shifts were corrected between conditions per subject, individual latency variability can still distort the condition effects. In a manner akin to Step3, we calculated the common median peak latency across all participants and further synchronized all trials to this benchmark. Subsequently, we were able to identify genuine amplitude effects that more accurately reflect the activation levels of brain activity. However, these differences may still include disparate spatial patterns. To assess the impact of scalp distributions, each trial was normalized by the global field power (GFP). GFP evaluates the overall intensity or magnitude of electrical activity by calculating the standard deviation of voltage values across all electrodes at each time point. This moment-by-moment normalization process ensures that the relationships between electrodes are preserved while scaling the amplitudes, referred to as profile analysis (McCarthy and Wood 1985). It enabled us to assess whether the topographic maps are identical between two conditions. By scaling the influence of activation levels through normalization, our focus is on examining the topographical disparities in decoding performance.

In the stepwise latency correction method using RIDE, various parameter settings are involved, including the time windows for component decomposition and the choice of a reference electrode for synchronization between conditions. In the context of investigating face priming effects in this study, in Step1 for decomposition, we selected time intervals of [0, 300] ms and [200, 900] ms for the S and C components, respectively, to narrow the search scope. For the R components, the decomposition window was set at [-300, 300] ms relative to the response. Each interval is assumed to cover the time span in which each component is anticipated to occur based on the visual examination of the averaged ERPs. In Step3, we utilized the ‘Pz’ electrode as the synchronization reference due to its prominent activity during the complex cognitive task. Nevertheless, it is worth noting that due to the effects of volume conduction, analyses using neighboring electrodes would yield similar patterns and in other paradigms, different electrodes may be the best.

## Results

In this section, we first consider the priming effects from the perspective of original averaged ERPs as a standard reference including all influences. Then we examine the impact of trial-to-trial variability on classifiers by analyzing changes in prediction accuracy, temporal decoding analysis, and saliency maps.

### Condition effects from the perspective of event-related potentials

By averaging single trials across conditions and participants, we quantified the original priming effects by examining the difference waveforms between primed and unprimed conditions (Fig. 2b). Based on latency, amplitude, and topography comparisons, we predefined two intervals of 120 ms each (indicated as shaded areas). The late priming effect (340–460 ms) encompassing the N400 component was identified, along with a later priming effect (580–700 ms), which has received less attention in prior work. These intervals of equal length were used for subsequent statistical analyses and comparisons of decoding performance. Specifically, the topographies showed centro-parietal positivities for the late priming effect, while the later priming effect had inverted polarities with centro-parietal negativities. To further examine these effects at the single electrode level, we focused on the ‘Pz’ electrode (refer to Fig. 4). The activity at this electrode exhibited contrasting amplitude differences across the intersection point. However, solely from the original ERPs, we cannot determine whether the primed or unprimed condition shows larger activations.

Next, we applied our framework to the original single-trial ERPs (Fig. 2b, Original). The decomposed clusters were shown in the supplementary material (Fig. S1). After we eliminated the S and R components (Fig. 2c, Step1) and corrected the within-condition jitter (Fig. 2d, Step2), the phase reversal was still present, resulting in ERP difference waves displaying two peaks and topographies with reversed polarities. These findings bore considerable resemblance to the original ERPs. It suggests that S and R components, as well as within-condition jitter, do not substantially impact the overall waveforms of priming effects, although there were some amplitude changes during these two steps. It also indicates that latency jitter has reduced the averaged ERP amplitudes, as evidenced by larger amplitudes in Step2 compared to Step1. This smearing of amplitudes renders suboptimal representations of condition effects.

However, by synchronizing latency shifts between primed and unprimed conditions based on the median peak latency at the ‘Pz’ electrode in Step3 (Fig. 2e), the phase reversal disappeared. Now, the topographies exhibiting similar, rather than reversed, polarities across these intervals suggest that the opposite polarities observed in the original ERPs were caused by latency shifts (cf. Fig. 1b Case 2). Additionally, the overall amplitudes and peak amplitudes of unprimed ERPs at the ‘Pz’ electrode were larger than those of primed ERPs (Fig. 4d, upper panel). Since this difference was not evident in the original ERPs (Fig. 4a, upper panel), it suggests that latency shifts can indeed distort amplitude differences. Latency shifts have diminished the amplitude differences between conditions. Furthermore, the two peaks seen in Step3 occurred earlier than in the preceding steps, as indicated by the increase in GFP before 200 ms. The observed phenomenon, arising from the fundamental distinction that the unprimed condition exhibited a larger and broader morphology in comparison to the primed condition, led to an earlier onset of the difference waveforms when aligning between conditions. Based on this, two topomaps from 260 ms to 380 ms and from 520 ms to 640 ms were displayed in the illustration for Step3, which showed increased parietal and central negativities, respectively, surrounded by occipito-temporal positivities. These results provide evidence for the effectiveness of our pipeline in uncovering genuine amplitude differences that might otherwise be obscured by latency shifts. However, although synchronization between conditions should result in only one peak for the priming effects, it actually extended over a long period from about 200 ms to more than 700 ms with two maxima at approximately 300 and 600 ms. This may suggest the existence of individual differences, which we addressed by synchronizing across participants in the subsequent step.

The results of synchronization across participants in Step4 are shown in Fig. S2, where the two peaks merged. When further normalized by the GFP (Fig. 2f, Step4), the amplitudes of the two conditions were scaled to the same overall activation level without altering the scalp topographies. On the magnitude scale of normalization, the temporal region still showed positivity, while the parietal area exhibited negativity. Comparing the topomaps between these two intervals further, we observed that the late priming effect had a larger difference compared to the very late priming effect, which was less prominent before normalization. The topomaps of difference scaled waveforms suggest that the activation level fluctuates based on this heterogeneity, indicating that the primed and unprimed conditions indeed have different topologies over time.

The data from one participant across the analysis steps were visualized separately for primed (Fig. 2g) and unprimed conditions (Fig. 2h). For each condition, the upper panels showed the distributions of reaction times (Original), latency lags (Step1 and Step2) and amplitude variabilities (Step3 and Step4), while the lower panels displayed the sorted trials at the ‘Pz’ electrode. The first panel shows the original ERPs sorted by RT. Priming decreases mean RT so that the RT distribution of the primed condition is shifted to the left relative to the unprimed condition. However, the latency distribution of C components cannot be observed in the original single-trial ERPs due to the unknown time markers and superposition of subcomponents. The disentanglement of amplitude and latency was visible after the elimination of S and R components when sorting the single trials according to C component latency lags (the second panel, Step1). By synchronizing the C component latencies within each condition (Step2), the latency shifts between conditions became evident in the middle panel. The last two panels focused on the amplitude variabilities at the ‘Pz’ electrode. Amplitude variability was calculated as the covariance between each single trial and its corresponding template electrode-by-electrode. After amplitude normalization, the amplitude variabilities were nearly zero. Collectively, our pipeline effectively disentangled latency and amplitude effects in a stepwise manner and the results provided evidence that the trial-to-trial variability indeed confounded condition effects from the angle of averaged ERPs.

## EEGNet assessment of condition effects by classification performance

In the previous section, we explored the impact of various variabilities on the condition effects of averaged ERPs. Inspired by this issue, we now aim to explore how these variabilities impact DNN-based classifiers, especially EEGNet in this paper. In this section, we evaluate the classification performance. If there is no significant change in prediction accuracy between consecutive steps, it would suggest that the variability eliminated in this step does not contribute to the classifiers’ performance.

**Fig. 3:**
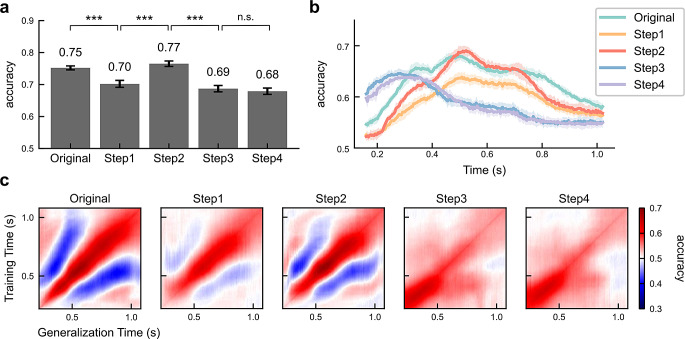
EEGNet assessment of condition effects. **a** The general classification performance of each step is depicted, where the models were trained on the whole epoch (1-second) from 100 ms to 1100 ms after target stimulus onset. The error bars represented the standard deviations across 10-fold cross validations. The prediction accuracies were statistically significantly better than chance (***: $\:p$<0.001, t-test; n.s.: not significant). The changes between consecutive steps were also statistically significant, except from Step3 to Step4. **b** Temporal decoding results. Within each step, the whole time epoch was divided into overlapping time segments, each with a duration of 120 ms. The 120-ms sliding window moved 4 ms each time. Each point is denoted by the endpoint of its corresponding segment. The shaded areas represented the standard deviations across 10-fold cross validations. All prediction accuracies were statistically significant ($\:p$<0.001, t-test). The prediction accuracy of the latency jitter-corrected trials (Step2) reached its highest level between approximately 500–600 ms, significantly surpassing the accuracy of the original single-trial ERPs. After synchronizing latency shifts between conditions (Step3), the depicted priming effects and associated prediction accuracies progressed forward in time and peaked around 300 ms. **c** The temporal generalization results represented the averaged prediction accuracies across 10-fold cross validations. The y-axis (training time) showed the time intervals used for training, while the x-axis (generalization time) displayed generalization using a specific model to test all segments. The red and blue regions indicated above-chance and below-chance accuracy, respectively. The below-chance accuracy resulted from latency shifts between conditions

We employed two different training strategies with distinct input lengths in our analysis. Firstly, we trained EEGNet to classify the whole epoch from 100 ms to 1100 ms after the target stimulus onset and evaluated the overall classification performance for different steps of latency control. The testing performance, depicted in Fig. 3a, was the mean prediction accuracy on testing datasets across the 10-fold cross validations. As compared to the chance level of 0.5 for a binary classification problem, all prediction accuracies were statistically significantly better ($\:p\:$< 0.001, t-test). The changes between consecutive steps were also significant, except between Step3 and Step4. However, relying solely on overall classification accuracy did not provide detailed insights into the specific characteristics of each step. To assess whether and when patterns were altered by the subsequent steps of latency control, we conducted the temporal decoding analysis. In each step, we analyzed time segments of 120 ms duration and slid the window by 4 ms over time. The model trained on one interval (*training time*) was then used to test all time segments in the epoch, which we refer to as the *generalization time*. The results of temporal decoding and temporal generalization analysis were presented in Fig. 3b and c, respectively.

To be more specific, for the original single-trial ERPs, the results showed that the prediction accuracy for the whole epoch reached 75%. The overall classification performance demonstrates that classifiers can reliably predict the priming condition in single trials due to inherent differences between these two processes. The temporal generalization results showed the above-chance predictions spread around the diagonal regions, rather than a square-shaped pattern suggesting consistent neural activity maintained over time. It suggests that adjacent time points exhibit shared but not identical patterns over a certain period. The occurrence of below-chance accuracy in the off-diagonal regions (indicated by the blue color, 500–1000 ms after stimulus onset) was associated with the little-studied later priming effect (> 500 ms). We hypothesized that the below-chance performance arose from a pattern reversal. The temporal generalization results provide convincing evidence that EEGNet is capturing a dynamic, evolving neural process with systematic latency shifts, as opposed to a sequence of discrete, static processing stages, in representing the priming effects during face recognition.

The accuracy in Step1 (71%) was slightly lower than that in Original and the significant drop suggests that the stimulus-related S component and response-related R component also play a role in discriminating the priming conditions. The related classification results of S and R components are shown in Fig. S3. Of particular importance in this context was the N250r component, which was initially observed in the original ERPs but was eliminated in Step1. Additionally, in the temporal generalization matrix, we still observed the presence of reverse patterns or below-chance accuracy, although they were somewhat attenuated. This finding suggests that the presence of response-related components with variable latency (e.g., reaction time) can also influence the reversed pattern and amplitude differences observed from the Original.

Variability in the timing of cognitive processes and their neural correlates across trials within the same participant can occur due to factors such as fatigue or momentary attention. When using a classifier that examines a fixed time window across numerous trials, it may struggle to accurately recognize latency jitter. Therefore, it is essential to correct the jitter to assess its true impact. Interestingly, the accuracy in Step2 after correcting within-condition jitter was even higher than the Original, reaching 77%. This finding suggests that jitter, as a form of variability, does not contribute positively to the discrimination task. Previous studies have suggested that the temporal convolution operations in the first block of EEGNet are robust enough to ignore minor variations, like latency jitter, to some extent (Solon et al. 2019). However, our results indicate that within-condition jitter plays a significant role in degrading performance in classifiers. The convolution operations do not completely consider this type of variability as meaningless noise but rather as features that need to be learned, despite their negative impact. Moreover, the temporal generalization results aligned closely with the Original. The below-chance accuracies still existed, although they were slightly less prominent than in Step1. This suggests that trial-to-trial jitter is not the main reason for the below-chance accuracy.

In Step3, after synchronizing within-subject latencies between conditions, the overall accuracy dropped to 69%, which indicates that priming-induced latency shifts play a crucial role in the measured priming effects. From the prediction accuracy on time segments over the time course (Fig. 3b), we observed that the priming effects started early. The previously observed reversed pattern disappeared, and above-chance accuracy was primarily found along the diagonal. Although previous studies have mentioned the phenomenon of off-diagonal elements with below-chance accuracy (Carlson et al. 2013; King et al. 2014), they did not employ operations to analyze it. Comparing this area from the Original to Step3 reveals that the off-diagonal regions result from priming-induced latency shifts. The later priming effect exhibits a temporal delay and pattern reversal relative to the earlier effect.

In Step4, as we further synchronized across participants and normalized amplitudes, the residual topographical distributions became the primary influencing factor. The general classification performance did not change significantly, indicating that the latency variability across subjects and the genuine activation levels may not contribute to classifiers. Although the magnitudes were scaled down, they did not significantly impact the discrimination ability. In the meantime, the significant prediction accuracy in Step4 demonstrates that the topographies of primed and unprimed ERPs differ and topographical heterogeneity has a crucial impact on discrimination. This is because if the topographies were the same, scaling would eliminate any difference. These results suggest that the classifier can learn temporal and spatial features from the patterns over time and correlations across electrodes, rather than relying solely on the actual activation levels. Besides, when comparing prediction accuracies across all steps, we noticed that Step2 achieved the highest accuracy. The reason behind this is that when within-condition jitter was eliminated, the differences between primed and unprimed conditions became more evident and pronounced.

## Saliency maps

**Fig. 4:**
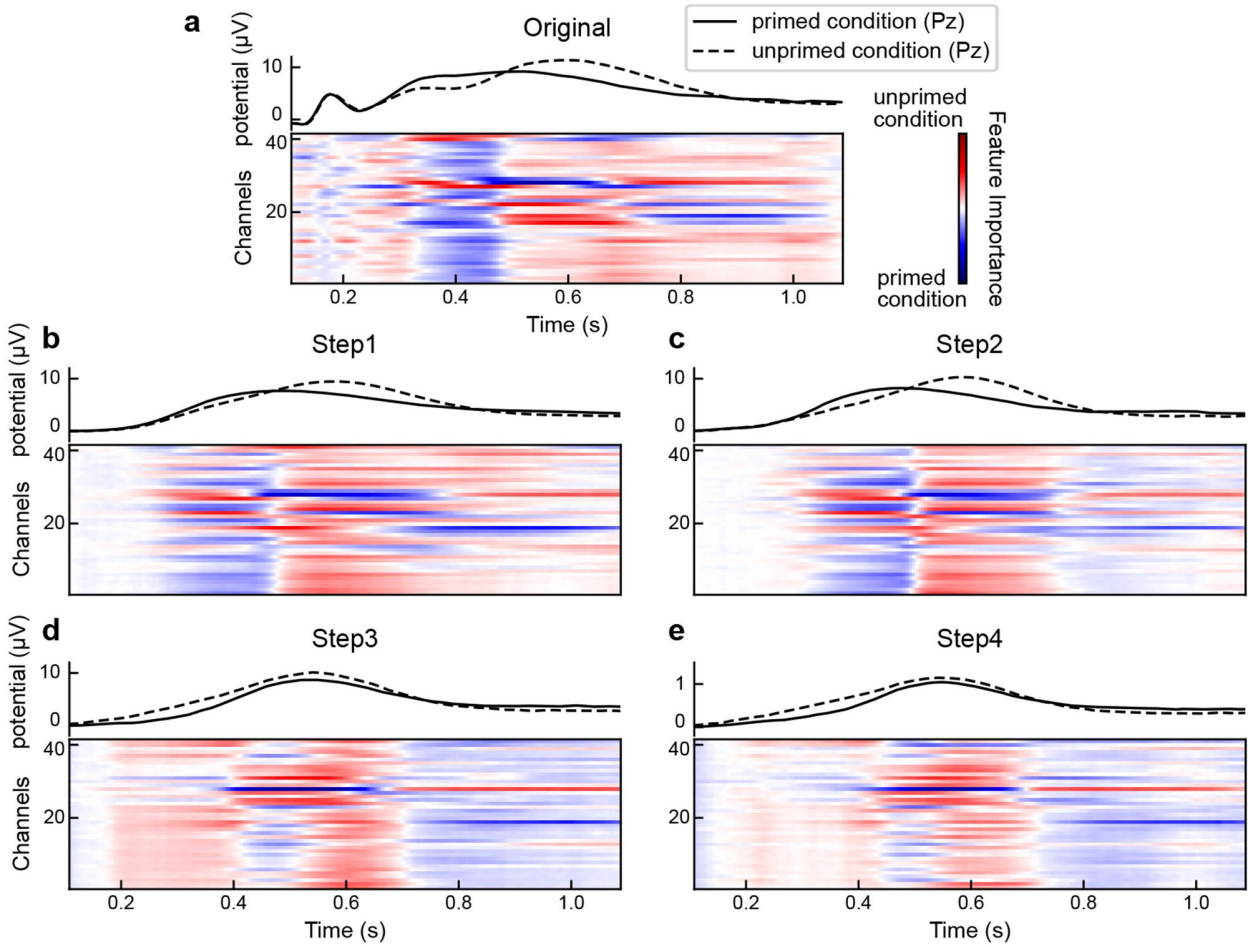
The saliency maps for discrimination between primed and unprimed conditions. **a–e** Original to Step4. The importance of features, considering both temporal and spatial aspects, is evident throughout the classification in the stepwise latency correction model. For consistent comparison, the darker blue and red highlight the more crucial features of the primed and unprimed conditions, respectively. The curves on the top panels demonstrate the brain activities at the ‘Pz’ electrode. We can observe two distinct intervals for primed and unprimed conditions until Step3. The findings suggest that priming effects originate from a single process with the latency shift, rather than distinct stages. In Step3, it is observed that the unprimed condition exhibits a wider and larger morphology compared to the primed condition, leading to the occurrence of another priming effect within the range of approximately 200 ms to 400 ms. This previously unidentified early priming effect, distinct from the N250r, becomes apparent only after correcting for amplitude differences caused by latency shifts

The purpose of this section is to employ interpretable artificial intelligence techniques to identify key elements during the classification process. We utilized the DeepLIFT approach of Shrikumar et al. (2017) to generate saliency maps. The saliency maps were calculated based on the model using the whole time epochs as inputs. In a binary classification task comparing conditions A and B, classifying condition A as A may be easier than classifying condition B as B in some cases, for example when condition A is more salient, acting as a clear signal amidst noise, while condition B is considered as ‘noise’. However, there are also instances where the opposite can be true, with condition B being more salient than condition A. The relative saliency of the two conditions can vary depending on the specific time windows and characteristics of the data being analyzed. We calculated the average feature importance for single trials that had a correct prediction probability greater than 0.9. Figure 4 displays the feature importance at each channel and time point across conditions. In the visualization for consistent comparison, darker red indicates that electrodes or time points are more critical and helpful in correctly identifying trials of unprimed conditions, while darker blue reveals more significant features in distinguishing primed condition trials.

Two distinct intervals playing a significant role in classifying primed and unprimed trials separately were observed. The interval (300–460 ms) represented the most crucial period for the primed (blue) condition. Interestingly, this interval occurred earlier than the interval (starting at about 450 ms), which was associated with the most important features of the unprimed (red) condition. The saliency map indicates that the important features related to the primed condition occur earlier compared to those associated with the unprimed condition, which is also consistent with the averaged ERPs where the primed condition exhibits larger and earlier fluctuations compared to the unprimed condition. This finding provides additional support for the presence of latency shifts between primed and unprimed conditions. When RIDE was applied in Step1 to remove the S and R components, the period (100–280 ms) no longer appeared to be important. This suggests that there are priming effects in the S components, such as the N250r component. After correcting for jitter in Step2, the boundaries between the two important intervals became more apparent. This indicates that the current features are more important and prominent in explaining why Step2 achieves the highest accuracy (cf. Figure 3a, b). Furthermore, the latency shifts persisted even after removing the S and R components (Step1) and within-condition jitter (Step2). However, when cognitive processes between conditions were further synchronized (Step3) and amplitudes were normalized (Step4), these two intervals merged due to the correction of latency shifts. As a result, clear boundaries between these two periods were no longer observable. All of these findings align with and provide a deeper understanding of the perspective derived from averaged ERPs, as demonstrated in the top panel that displays the primed and unprimed ERPs recorded at the ‘Pz’ electrode (Fig. 4).

In addition to yielding consistent results, these findings provide novel insights that go beyond what can be observed from averaged ERPs. The presence of two distinct intervals in the Original raises the question of whether primed and unprimed conditions involve different processes or a single process with latency shifts. Determining this distinction solely from averaged ERPs or the saliency maps of the original single-trial ERPs is challenging. However, by correcting for latency shifts between conditions, the two intervals merged, suggesting that priming effects mainly arise from a single process with latency shifts rather than two distinct processes. Additionally, in Step3, the unprimed condition exhibited a broader and larger morphology compared to the primed condition (Fig. 4d, upper panel), resulting in an additional priming effect (approximately 200–400 ms). This early priming effect which was previously unidentified (not the N250r), became evident only after correcting for amplitude differences induced by latency shifts.

## Discussion

Data-driven models have gained increasing attention in current research within the field of cognitive neuroscience. Most DNNs primarily focus on voltage values of single trials. Without taking domain knowledge of signal attributes into consideration, DNNs do not explicitly measure the properties of amplitude and latency of single trials. In this paper, we applied a stepwise latency correction model to control for different types of variability. To measure the significance of each factor, we used data at each step from all participants and conditions to train models. By comparing the same evaluation metric, such as prediction accuracy or saliency maps, we could assess the contribution of variability to the classification process at each step. Analogous to condition effects, our pipeline effectively detected – by elimination – the impact of stimulus- and response-locked components, latency jitter, latency shifts, latency-corrected amplitudes, and general amplitude differences on classifier performance. As a comparison, we also showed that the relationship between latency and measured amplitude can introduce confounding factors in the analysis of averaged ERP components.

### Generalizability across different modeling approaches and comparison with other latency correction methods

The benefits of latency correction models are consistent across different modeling approaches. Once the step-wise latency correction can be applied to the components of interest in a given task, the traditional machine learning methods (e.g., logistic regression (Hastie et al. 2009)) or other deep neural networks (e.g., recurrent neural networks (Elman 1990)), which have been well established in the literature can then be applied. We showed that the pattern of classification accuracy and related temporal decoding across consecutive steps (Fig. S4) mirrors that of EEGNet. Meanwhile, it’s essential to recognize that the specific configurations and parameters, such as the time windows for component decomposition and the selection of reference electrodes, must be customized based on the research objectives and the particularities of the EEG data under examination.

On the other hand, there are many other algorithms handling latency variability based on filtering (Cerutti et al. 1987; Jaśkowski and Verleger 1999; Aniyan et al. 2014) or classifiers (Hu et al. 2011; Solon et al. 2019). However, these methods do not decompose ERPs into different component clusters so their estimated latencies are a mix of stimulus-, cognition-, and response-related temporal information. And our method also demonstrated its effectiveness and superiority, e.g., with respect to low signal-to-noise ratio (Ouyang et al. 2011, 2015a, 2017; Ouyang and Zhou 2020).

### Generalizability across different cognitive tasks

Although in this paper, we used face repetition priming as a test case and employed EEGNet as the classifier to discriminate single trials into primed or unprimed conditions, it is important to note that our framework can be applied to any classifier, and any experiment that involves latency-varying signals, making it adaptable to a wide range of cognitive studies. As demonstrated in our previous research works (Ouyang et al. 2011, 2015a, 2017; Ouyang and Zhou 2020), decomposing ERPs into different component clusters and correcting for within-condition latency variability have been effectively applied to data from various paradigms such as word recognition and go/no-go tasks, etc. Different research groups have applied the method to different tasks and experiments, such as language processing, cognitive conflicts, and task switching (Petruo et al. 2021). However, it’s important to note that different tasks and components may exhibit different degrees of latency variability and latency shifts. Thus, the outcomes can diverge across different steps, potentially differing from the face repetition priming case presented in the present paper.

### Latency jitter may not be noise in cognitive processes

The jitter-induced amplitude smearing can reduce classification accuracy. While within-condition jitter is commonly viewed as noise when analyzing averaged ERPs (Ismail Fawaz et al. 2019), our findings suggest that it should not be just considered as meaningless noise of brain activity. Although the operations of DNNs, like temporal convolution operations of CNNs, are robust in mitigating minor variations to some extent, the jitter survives after convolution and has a negative impact on the performance of classifiers. Therefore, jitter can be considered as a source of noise that confounds amplitude differences and also as a signal of interest that reflects important characteristics of cognitive processes. Previous studies investigating the variability of brain signals within individual have demonstrated significant associations with brain function, development, and task performance (Garrett et al. 2013; Rostami et al. 2017). Our results further support this claim from the perspective of classifiers. However, further exploration is necessary to fully understand how this variability is linked to brain function, particularly through the assessment of individual differences.

### New insights for priming community

In previous face cognition studies, two prominent priming effects, the N250r, and the N400, have been identified in the averaged ERPs of difference waveforms between primed and unprimed conditions (Schweinberger et al. 1995, 2002; Herzmann et al. 2004; Herzmann and Sommer 2007; Wilhelm et al. 2010; Kaltwasser et al. 2014; Weller et al. 2024). However, these effects are typically measured before the intersection. It is worth noting that another priming effect is often observed around 600 ms or later, after the intersection, but this effect has found little attention in previous studies. The present study reveals that latency shifts between conditions can result in artefactual amplitude differences. In the meantime, latency shifts have a significant positive contribution which may be helpful for certain purposes. The prevailing understanding of how shifts in latency between conditions can boost the performance of classifiers is that these shifts in timing often reflect the different time demands of the cognitive or mental processes involved. If any factor, such as task difficulty or aging, slows down or speeds up specific mental processes (e.g., memory retrieval or response selection), it is reflected in corresponding latency shifts in ERP components. The present approach might help uncover such changes in cognitive speed and disentangle them from amplitude differences. We recommend that future research in the priming community takes into consideration these latency shifts and the part of the waveform after the crossing point for a more comprehensive understanding of priming effects.

### Get inspiration from domain knowledge and go beyond

Many DNN-related studies aim to enhance the accuracy of predicting EEG single trials by improving the model’s architecture. However, as the algorithm’s complexity increases, it becomes difficult to identify neurophysiologically meaningful features, particularly when dealing with uncommon paradigms or effects linked to higher-level cognitive processes. Various post-hoc interpretability techniques, such as Deconvolution (Mahendran and Vedaldi 2016), Integrated Gradients (Sundararajan et al. 2017), and GradCAM (Selvaraju et al. 2017), play a crucial role in understanding the outcomes of neural networks. Nevertheless, when applied to a specific task, these techniques yield different results, leading to the question of identifying the most suitable method for ensuring accurate post-hoc interpretability. While certain metrics can help evaluate the reliability of each method (Turbé et al. 2023), our findings indicate that consistent observations between empirical studies based on domain knowledge and data-driven approaches can also aid in the interpretation process. In the meantime, this consistency can also serve as a foundation for gaining new insights that go beyond what empirical studies alone can reveal.

## Conclusion

Taken together, these results indicate that DNN-based classifiers can assess changes in voltages resulting from underlying latency and amplitude modifications and detect significant features in latency observed in the evoked responses. In other words, classifiers can extract the inherent characteristics through single-trial analysis, implicitly learn about latency and corresponding variability, and incorporate these properties into their decision-making process. However, these features cannot be explicitly identified and explained. Only by explicitly decoupling the amplitude and latency information from domain knowledge, we are able to more accurately measure these properties and assess the impact of trial-to-trial variability on classifiers.

### Limitations and outlooks

We have made attempts to analyze the temporal and spatial kernels directly, but we have not yet discovered reliable explanations for their neurophysiological meanings due to the complexity of cognitive process. This area needs further exploration in future research. Additionally, both latency and amplitude measurements, along with their variability, depend on the specific template morphology for each subject and condition, as determined by participant-based RIDE. We used the ‘Pz’ electrode as a reference to synchronize the remaining electrodes by an equal extent. However, we have not yet taken into account latency variations between different electrodes such as those caused by noise or time-shifted overlap of brain-electrical sources. Therefore, it is necessary to investigate whether latency jitter, latency shifts, latency-corrected amplitude, latency variations between electrodes, or other forms of variability contribute to subject-based classifiers through individual differences studies. How the analogy between deep neural networks and traditional methods from empirical studies, as well as the general domain knowledge from hypothesis-driven studies can facilitate us to study brain-behavior relationships also needs further investigation. Moreover, it’s conceivable to integrate the stepwise latency correction method into real-time EEG analysis pipelines, for example as an online measure for the latency of brain responses (e.g. in training mental speed). This kind of integration could be beneficial for applications like brain-computer interfaces (BCIs), which will be one of our future research aims.

## Electronic supplementary material

Below is the link to the electronic supplementary material.

Supplementary Material 1

## Data Availability

Data and code will be made available based on reasonable requests.
