# Supplementary material for: Assessing the influence of latency variability on EEG classifiers - a case study of face repetition priming
Source: Cogn Neurodyn. 2024 Oct 21;18(6):4055–69. doi: 10.1007/s11571-024-10181-2 (PMC11655819; doi:10.1007/s11571-024-10181-2)
Supplement: Supplementary file 1 — Supplementary Material 1 [file 11571_2024_10181_MOESM1_ESM.pdf]

Title

Assessing the influence of latency variability on EEG classifiers - A case study of face repetition priming

Authors

Yilin Li[1,2,3], Werner Sommer[1,5,6,7,*], Liang Tian[1,4,8,*], and Changsong Zhou[1,3,4,5,*]

Affiliations

[1]Department of Physics, Hong Kong Baptist University, Kowloon Tong, Hong Kong SAR, China

[2]Institute of Interdisciplinary Studies, Hong Kong Baptist University, Kowloon Tong, Hong Kong SAR, China

[3]Centre for Nonlinear Studies and Beijing-Hong Kong-Singapore Joint Centre for Nonlinear and Complex Systems (Hong Kong), Hong Kong Baptist University, Kowloon Tong, Hong Kong SAR, China

[4]Institute of Computational and Theoretical Studies, Hong Kong Baptist University, Kowloon Tong, Hong Kong SAR, China

[5]Life Science Imaging Centre, Hong Kong Baptist University, Kowloon Tong, Hong Kong SAR, China

[6]Department of Psychology, Humboldt-Universität zu Berlin, Berlin, Germany

[7]Faculty of Education, National University of Malaysia, Kuala Lumpur, Malaysia

[8]Institute of Systems Medicine and Health Sciences, Hong Kong Baptist University, Kowloon Tong, Hong Kong SAR, China

*Corresponding Author:

Werner Sommer: werner.sommer@cms.hu-berlin.de

Liang Tian: liangtian@hkbu.edu.hk

Changsong Zhou: cszhou@hkbu.edu.hk

Declarations

Acknowledgments: The authors would like to thank Ouyang Guang for his helpful discussions on RIDE applications. This work was supported by the Hong Kong Research Grant Council (Nos. GRF 12200620, GRF12201421, and CRF C2005-22Y), the National Natural Science Foundation of China (Nos. 11975194, 12275229), Hong Kong Baptist University Initiation Grant for Faculty Niche Research Areas (RC-FNRA-IG/23-24/SCI/05), and the German Research Foundation (Deutsche Forschungsgemeinschaft; HI 1780/2-1 & SO 177/26-1).

Conflict of interest: The authors have no relevant financial or non-financial interests to disclose.

Data and code availability: Data and code will be made available based on reasonable requests.

Supplementary Information

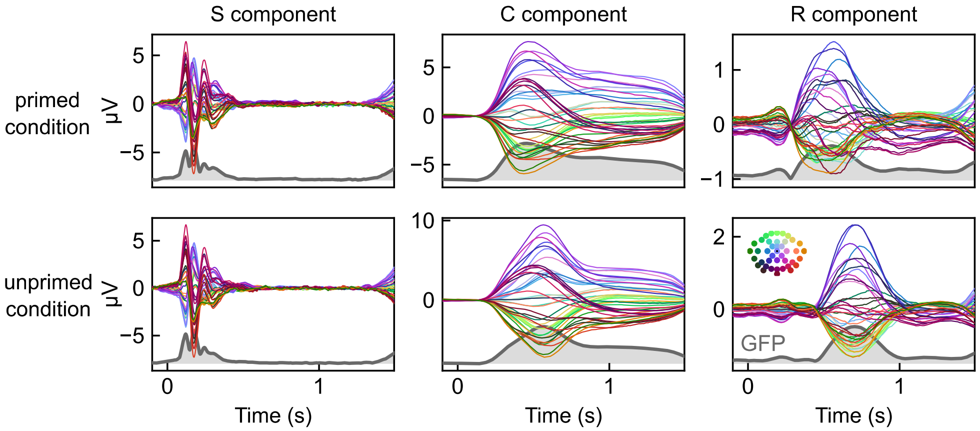

Fig. S1 Decomposition results by RIDE. The top and bottom panels represent the decomposition results for primed and unprimed conditions, respectively.

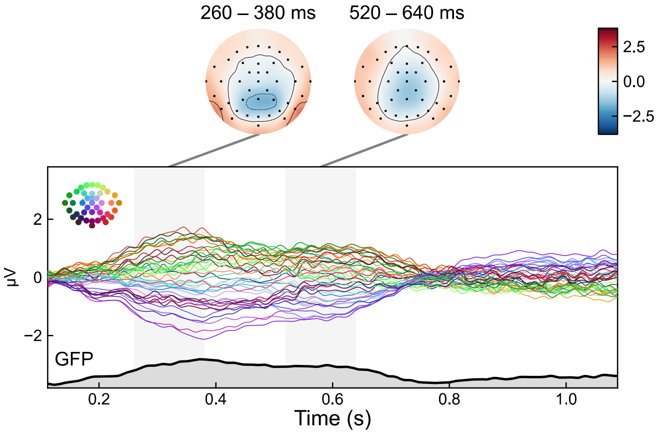

Fig. S2 Averaged ERPs of difference waveforms between primed and unprimed conditions after synchronizing latency shifts across participant groups (Step4).

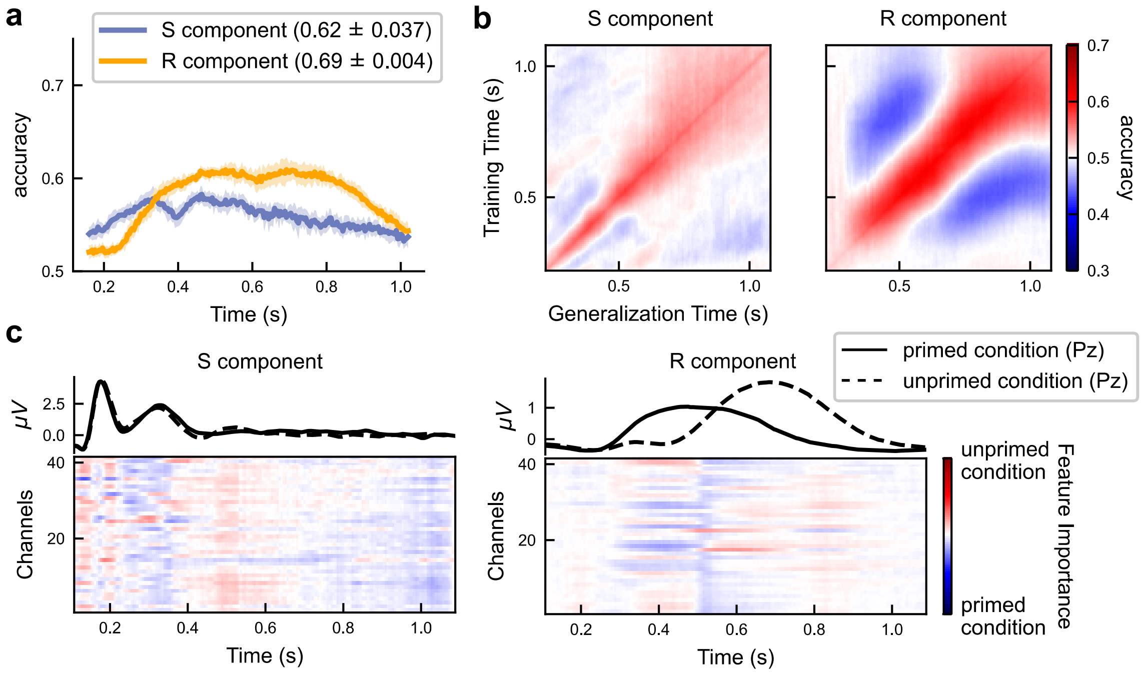

Fig. S3 EEGNet assessments of S and R components. **a** Classification results. The curves display the temporal decoding results and the legends indicate the general prediction accuracies along with mean values and standard deviations across 10-fold cross-validations. **b** Temporal generalization results. **c** Saliency maps.

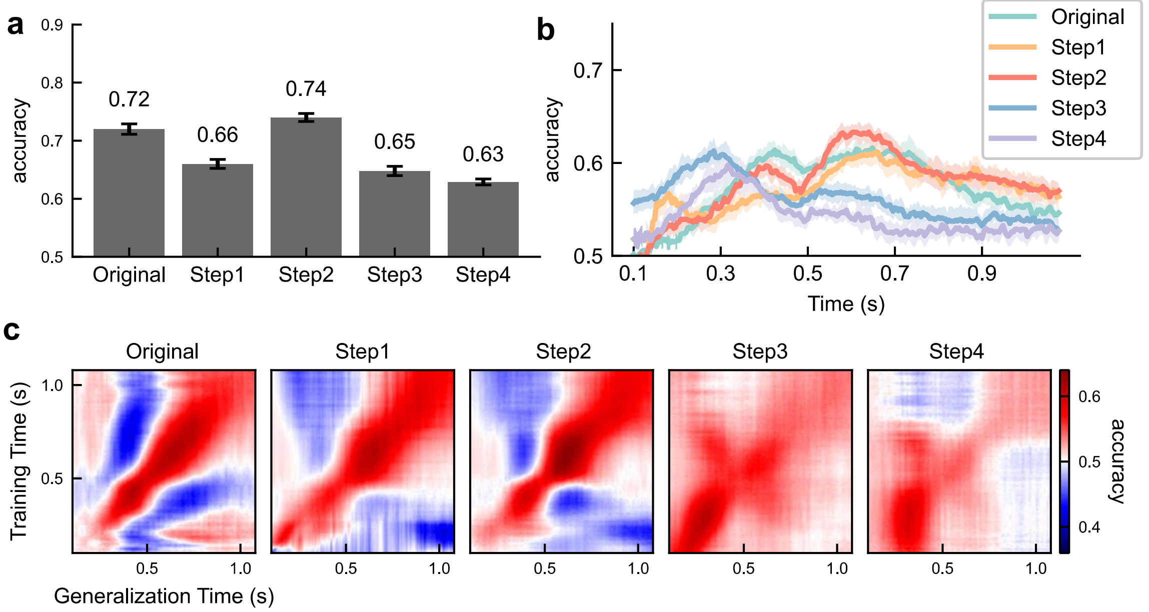

Fig. S4 a RNN classification results based on whole time epochs. **b** Temporal decoding results using logistic regression trained on each time point. **c** Temporal generalization results using logistic regression trained on each time point.
